# Mapping global value chains at the product level

**DOI:** 10.1140/epjds/s13688-025-00521-5

**Published:** 2025-03-12

**Authors:** Lea Karbevska, César A. Hidalgo

**Affiliations:** 1 Center for Collective Learning, IAST, Toulouse School of Economics, 1 Esplanade de l’Universite, Toulouse, 31080 France; 2 SCAIL, University of Cambridge, 17 Charles Babbage Rd, Cambridge, CB3 0FS United Kingdom; 3 Center for Collective Learning, CIAS, Corvinus University of Budapest, Közraktár u. 4-6, Budapest, 1093 Hungary

**Keywords:** Global value chain, Products, Network, Supply chain, Trade

## Abstract

Value chain data is crucial for navigating economic disruptions. Yet, despite its importance, we lack publicly available product-level value chain datasets, since resources such as the “World Input-Output Database”, “Inter-Country Input-Output Tables”, “EXIOBASE”, and “EORA”, lack information about products (e.g. Radio Receivers, Telephones, Electrical Capacitors, LCDs, etc.) and instead rely on aggregate industrial sectors (e.g. Electrical Equipment, Telecommunications). Here, we introduce a method that leverages ideas from machine learning and trade theory to infer product-level value chain relationships from fine-grained international trade data. We apply our method to data summarizing the exports and imports of 1200+ products and 250+ world regions (e.g. states in the U.S., prefectures in Japan, etc.) to infer value chain information implicit in their trade patterns. In short, we leverage the idea that due to global value chains, regions specialized in the export of a product will tend to specialize in the import of its inputs. We use this idea to develop a novel proportional allocation model to estimate product-level trade flows between regions and countries. This contributes a method to approximate value chain data at the product level that should be of interest to people working in logistics, trade, and sustainable development.

**Supplementary Information:**

The online version contains supplementary material available at 10.1140/epjds/s13688-025-00521-5.

## Introduction

Value chain data is important to understand the resilience and systemic effects of disruptions, such as natural disasters (Park et al. [35], Abe and Ye [1]), climate change (Ghadge et al. [13]), war (Ruta [30], Ali et al. [2], Laber et al. [24]), and disease (OECD [34]). Publicly available value chain data, however, such as the Organisation for Economic Co-operation and Development (OECD) Inter-Country Input-Output Database (OECD [33]), the World Input-Output Database (Timmer et al. [41]), EXIOBASE (Stadler et al. [40]), and EORA (Lenzen et al. [26, 27]) have limited sectoral resolution, being often disaggregated into a few dozen industries. This limited granularity is inadequate for applications where detailed product or sectoral resolution is needed, such as identifying critical industries, developing strategies and tracing the environmental impact of products in a period of political, health and weather uncertainties (Johnson [21], Diem et al. [10]).

Unlike value chain data, international trade data is much more granular, with over 5000 categories at the “six-digit level” (Harmonized System 6 (HS6) (Chaplin [8])) and over 1000 categories at the “four-digit level” (Harmonized System 4 (HS4)). Yet, while international trade data is also a go-to dataset for analysts working to understand disruptions, trade data lacks explicit information about value chain relationships. Trade data tells us that China imports iron ore from Brazil, but it does not tell us what that iron ore is used for (e.g. cars, iron rods, aircraft, etc.). But can we use granular international trade data to estimate product-level value chain relationships? Trade theory tells us that trade data must contain implicit information about value chain relationships. This information should be hidden in a country or region’s specialization patterns and we should be able to extract it by using trade theory-inspired features combined with machine learning techniques.

The idea of mapping value chains from trade data, however, is not new. Several projects have tried to combine input-output tables (Leontief [28]) and trade data in efforts to map global value chains (Lenzen et al. [26, 27], Timmer et al. [41], OECD [33]). These efforts use national input-output tables, connecting sectors at the local level with trade data, to estimate the volume of imported inputs used in each sector of an economy. These efforts, tend to rely on proportional allocation methods, where imports are distributed among sectors in the same proportion as local inputs. That is, they assume, for instance, that if 20% of the steel produced in a country is used for the production of machinery, then 20% of the steel imported from any country will also be used for the production of machinery. The result datasets (Lenzen et al. [26, 27], Timmer et al. [41], OECD [33]), however, still have a limited granularity and could benefit from increased sectoral and spatial resolution.

The use of machine learning for mapping supply chains has also been explored in several efforts. This includes supervised machine learning models capable of predicting firm-level supply chains (Mungo et al. [32]). Yet despite achieving high accuracy, these models often grapple with limitations such as reliance on sector-specific data (e.g., automotive (Kosasih and Brintrup [22]), energy (Kosasih et al. [23]), aerospace (Brintrup et al. [7])), country-specific data (e.g., the United Kingdom (U.K.), Japan (Mori et al. [31]), or South Korea (Lee and Kim [25])), and a notable absence of product-level information.

In this study, we introduce a value chain mapping method designed to estimate input-output relationships at the product level. Our approach involves fine-tuning the model on trade data between countries and subsequently producing the results on trade data between regions. Notably, this method after being successfully fine-tuned, can also be extended to estimate input-output product relationships between individual firms.

Detailed value chain data is crucial for a number of applications, exemplified by the following four instances.

First, consider the disruptions caused by the Ever Given, the massive container ship that in 2021 became stranded in the Suez Canal (BBC [5]). By blocking the Suez Canal, the Ever Given impeded the global flow of products, including oil, robusta coffee beans, furniture, and retail, between Asia and Europe (Martin [29], Domonoske [11]). The resultant scarcity of coffee beans, for example, had a cascading effect throughout the value chain, hampering the production of instant coffee. The Ever Given incident underscores the critical role of detailed value chain data in understanding and mitigating the impact of supply chain disruptions on various industries.

Second, value chain data can inform us about questions with geopolitical implications. Countries often avoid sourcing key components from geopolitical rivals. For instance, by organizing their value chains to avoid depending on potential enemies for strategic resources, such as fuel or electronics.

Third, value chain data can be a key input for environmental assessments (Stadler et al. [40]), since it is needed to account for the environmental impact of imported goods.

And finally, value chain data can be important to those working on corporate ethical responsibility. For instance, a clothing company may want to have traceability of its inputs to ensure its products are not produced using forced or child labour.

Yet, despite the glaring need for value chain data, there are no granular publicly available value chain datasets with fine spatial and sectoral resolution. In this paper, we explore the creation of a method to infer value chain relationships from international trade data in an effort to create product-level maps of global value chains.

In brief, our method exploits the idea that geographies that specialize in the export of a product will tend to specialize in the procurement of its inputs (Hummels et al. [20], Timmer et al. [42], Constantinescu et al. [9]). This tendency should be observed twice in trade data: upstream and downstream. The upstream tendency should be expressed in the products imported by a location that specializes in the export of a product. That is, we expect exporters of computers to specialize in the import of Liquid Crystal Displays (LCDs). Similarly, the downstream tendency should be expressed in the products exported by a location specialized in the import of a product. That is, importers of LCDs will tend to specialize in the export of computers. Here we combine both upstream and downstream specialization patterns in a model that we optimize to identify input-output relationships. We apply this model to a dataset summarizing the exports and imports of 1200+ products and 300+ world regions (e.g. states in the United States (U.S.), prefectures in Japan, etc.) to create a product-level dataset of value chain relationships.

Our method, however, is not without limitations. While it is designed to operate at the product level, it is not perfectly accurate, meaning that it provides some false-positive relationships. Also, it does not provide a full input-output network, but a set of the most likely value chain links for each product. Moreover, our method requires optimizing four different parameters, a process that can be slow and complicated.

Despite this limitation, we find that using trade theory inspired features results in a model that correctly identifies 70% of the first input for machinery products, validating the possibility of using international trade data at the regional level to identify value chain relationships.

In the remaining sections of this paper, we provide a detailed description of our data and methods. The structure of the paper is as follows: we begin with a section that discusses the data utilized in our model (section *Data*). First, we introduce some of the essential trade theory concepts that underpin our approach (section *Trade Theory*). Then we use these concepts to develop a proportional allocation model to assign trade flows along a value chain (section *Proportional Allocation Model*). Next, we introduce our “Backward & Forward” method to predict input-output relationships between products (section *Methodology*) and use it to construct a product-level dataset (HS4 and HS6 level) fine-tuned on the OECD Inter-Country Input-Output table (OECD [33]). We then manually validate a random sample of the results obtained by the “Backward & Forward” method to estimate its accuracy. Lastly, we explore three applications: estimating trade flow between regions and input-output products, extending the results beyond three inputs and assessing the average complexity index of products (Hausmann et al. [15]) based on their position in the value chain (section *Implications*). Our findings contribute to the development of computational methods aimed at constructing global value chain datasets.

## Data

We leverage fine-grained international data compiled by the Observatory of Economic Complexity (Simoes and Hidalgo [38]) (oec.world) spanning from the year 2017 to 2020. This is data on exports and imports at the regional level for 5890 HS6 products. Because of incompatibilities in data reporting (not all countries report regional trade data using the same classification), our sample is limited to regional data from 8 countries: Brazil (32 regions), Canada (13 regions), Chile (16 regions), China (31 regions), Japan (42 regions), Russia (85 regions), Spain (53 regions) and the United States of America (54 regions).

We clean this dataset by removing unknown regions and reexports such as “Reexportação”, “Exterior”, “Mercadoria Nacionalizada”, “Não Declarada” and “Consumo de Bordo” from the data of Brazil, “Unknown” from the data of the U.S. and Japan and “Sin provincia asignada” from the data of Spain. This leaves us with 318 regions.

**Figure 1:**
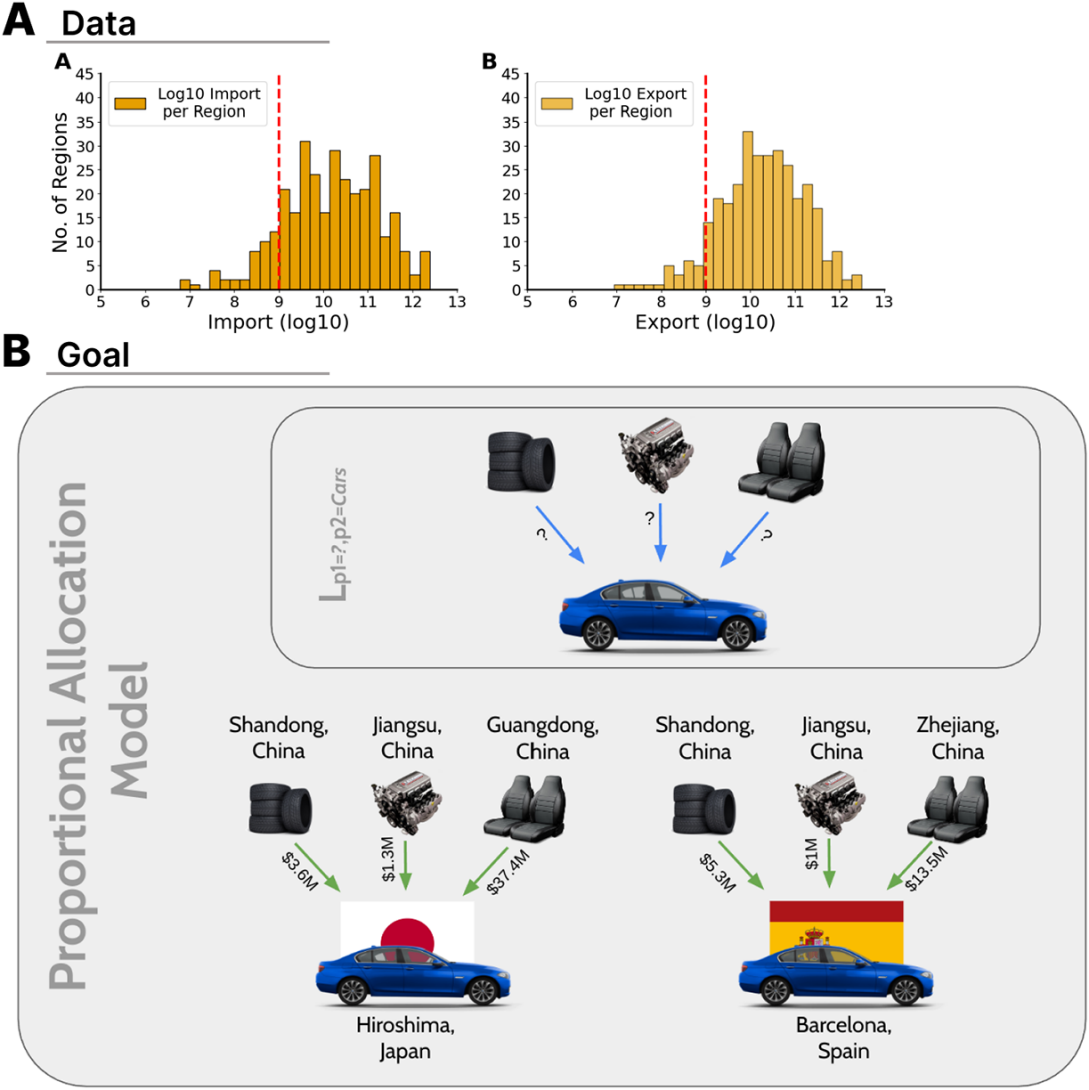
(A) Total import (a) and export (b) distributions across 9 regions from 2017-2020 from the Observatory of Economic Complexity data. Regions include Brazil, Canada, Chile, China, Japan, Mexico, Russia, Spain, and U.S.. (B) The proportional allocation model utilizes the input-output relationship produced by the L term and further quantifies the trade flow between two regions. In this figure, the term L provides the information that Cars need Rubber Tires, Engine Parts and Seats for their production. Whereas, the proportional allocation model uses the input-output information to quantify the exports of Rubber Tires, Engine Parts and Seats from Hiroshima to Barcelona which would be used for Barcelona’s local production of Cars

We then remove small regions that tend to have noisy signals about exports and imports (a few dollars of exports and imports can drastically change the observed specialization pattern of regions with low trade volumes) (Hidalgo [16]). After inspecting the distribution of exports and imports (Fig. 1), aggregated from 2017 to 2020, we remove regions on the left tails of the export’s and of the import’s distributions. These are regions which in these four years imported or exported in total less than 1 billion United States dollar (USD) (e.g. Ivanovo Region, De Magallanes Y Antartica Chilena, Paraíba, etc.). After removing these 49 regions we are left with a final sample of 269 regions.

We note that our data contains export and import information between regions and countries. That is, we know what Barcelona imports from Brazil, or what Sao Paulo imports from Spain, but not what is traded between Barcelona and Sao Paulo.

Finally, we map the HS6 product codes with their respective HS4 codes and names, and are left with 1230 HS4 and 5890 HS6 unique product categories.

In addition, we use the 2021 edition of OECD Inter-Country Input-Output (ICIO) data to fine-tune our model. This is a table containing 45 unique industries based on ISIC Revision 4 (industry, not product categories) for 66 countries. From this data, we produce two tables: OECD specialization and the OECD labeled data. A description of this data can be found at OECD [33]. For further details on the data manipulation see Additional file 1.

## Trade theory

Trade theory is a branch of economics focused on regional and international trade. A key contribution goes back at least 200 years to the work of the British economist David Ricardo [36]. In this paper, we use Ricardo’s concept of *comparative advantage* to create some of the features used in our model.

A location is said to have *comparative advantage* in the products that it is specialized in. Trade theory, in particular, the Heckscher-Ohlin model (Flux [12]), tells us that *comparative advantages* inform us about the factors that an economy is well endowed with. For instance, we expect economies endowed with vast maritime resources to specialize in the exports of fish and landlocked mountainous economies to specialize in the exports of minerals.

In today’s globalized economy, however, where intermediate inputs are highly mobile, economies often specialize in processes that are not necessarily pinned down by the presence of natural resources but by the availability of knowledge (Hausmann et al. [14]). That is, countries that export cars or furniture do not do so because they are endowed with iron or lumber (they can source these from global markets) but because they are equipped with skilled labour, technological expertise, and efficient manufacturing infrastructure. This means that countries and regions will tend to import some of the inputs they need to produce the outputs they export. Thus, we should be able to observe value chains implicitly, albeit imperfectly, in international trade flows.

To estimate *comparative advantages* in practice scholars use indicators of Revealed Comparative Advantage (RCA) (Balassa [4]) (also known as the Location Quotient in urban economics).

Formally, the Revealed Comparative Advantage of a location in an activity is the ratio between observed and expected exports that can be obtained by simply double-normalizing the export matrix. That is, the $RCA$ of a location *l* in a product *p* is:

1 $$\begin{aligned} RCA_{lp} &= \frac{X_{lp}}{ \sum _{p'} X_{lp'}} / \frac{\sum _{l'} X_{l'p}}{\sum _{l'p'} X_{l'p'}}, \end{aligned}$$

where $X_{lp}$ are the exports of location *l* in product *p*.

When a location has an RCA larger than 1 in a product, we say that the location is specialized in that product since it exports more than what it is expected for a location of the same size and for a product with the same global market.

Going forward, we define two versions of $RCA$. An export $RCA^{export}$, as defined in equation (1), and an import $RCA^{import}$ defined in the same manner, but where $X_{lp}$ represents the imports of location *l* in product *p*. The $RCA^{import}$ should tell us about the product that a region imports too much of. Our hypothesis is that by exploiting specialization patterns across multiple geographies we can generate features that when fed into a machine learning model can recover information about global value chains.

## Proportional allocation model

Formally, our goal is to estimate the tensor $X_{r_{1} p_{1} r_{2} p_{2}}$. This tensor represents the flow of product $p_{1}$ coming from region $r_{1}$ and used in region $r_{2}$ to produce product $p_{2}$. The data we have available, however, is more incomplete and represents two aggregates of the aforementioned tensor. These are: $X_{r_{1} p_{1} c_{2}}$ and $X_{c_{1} p_{1} r_{2}}$, which denote, respectively, the exports of product $p_{1}$ by region $r_{1}$ to country $c_{2}$ (where region $r_{2}$ is located) and the imports of product $p_{1}$ by region $r_{2}$ coming from country $c_{1}$ (where region $r_{1}$ is located).

We can estimate the flow value in product $p_{1}$ from region $r_{1}$ for the production of $p_{2}$ in region $r_{2}$ ($X_{r_{1} p_{1} r_{2} p_{2}}$) using the following proportional allocation model:

2 $$ X_{r_{1} p_{1} r_{2} p_{2}}= \frac{X_{r_{1} p_{1} c_{2}}}{\sum _{r_{1} \in c_{1}}X_{r_{1} p_{1} c_{2}}} \underbrace{L_{p_{1} p_{2}}}_{\text{unknown}} \frac{X_{r_{2} p_{2}}}{\sum _{p_{2} } X_{r_{2} p_{2}}L_{p_{1} p_{2}}} X_{c_{1} p_{1} r_{2}}. $$

Here the first fraction represents the share of exports of product $p_{1}$ by country $c_{1}$ coming from region $r_{1}$ and going to country $c_{2}$. For example, the share of Tokyo in Japan’s exports of semiconductors to Spain.

The second term, $L_{p_{1} p_{2}}$ is a binary matrix where 1 represents an input-output relationship between products $p_{1}$ and $p_{2}$ (Fig. 1). It will be the main challenge of our estimation method.

The third term is the share of exports of product $p_{2}$ by region $r_{2}$ over all of region’s $r_{2}$ exports that use $p_{1}$ as an input. For example, Madrid’s share of car exports over all products that use semiconductors as an input.

Finally, the term ($X_{c_{1} p_{1} r_{2}}$) represents the exports of product $p_{1}$ flowing from country $c_{1}$ to region $r_{2}$.

In principle, we can use trade data to estimate all of the terms of this equation except for the matrix *L*. Put together, equation (2) provides a proportional allocation model to estimate product specific value chain trade flows between a pair of regions.

Our next goal, is therefore, to develop a method to estimate $L_{p_{1} p_{2}}$.

## Methodology

### Backward & forward method

A link in a value chain can be traversed in two directions: a downstream or forward direction (from sunflower seeds to sunflower oil) and an upstream or backward direction (from sunflower oil to sunflower seeds) (Singer and Donoso [39]).

To estimate the term $L_{p_{1} p_{2}}$ from equation (2) we introduce the “Backward & Forward” method. The “Backward & Forward” method combines downstream and upstream value chain flows.

In the “Forward” approach, we start by selecting an import product $p_{1}$ and then we select the regions that import a disproportionately large amount of product $p_{1}$ (using an $RCA^{import}$ measure). This provides us with a list of locations sorted by import RCA (e.g. Alabama, Aguascalientes, etc.), which are places that import “too much” of that product (e.g. engines, batteries, etc.). We then look at the export specialization of these regions. The result is a matrix of the exports of the locations that import “too much” of product $p_{1}$. We then try to learn the outputs (e.g. cars, motor vehicles, etc.) associated with the import from the over-expressed exports of these locations.

In the “Backward” approach we first select an export product $p_{2}$ and then identify the locations that export a disproportionately large amount of product $p_{2}$. We then analyse what these locations specialize in, in terms of imports. The result is a matrix of the imports of the locations that export “too much” of the selected product. We then use this method to learn the inputs of product $p_{2}$.

We note that every product has an input but not every product has an output. For example, “Rolled Tobacco” (a.k.a. cigarette) is a final product that goes directly into consumption. While raw materials such as “Iron Ore” still need excavation machines to be extracted and transported. For that reason, we identify inputs of every product by first applying the “Backward” and then validating with the “Forward” approach. We call this the “Backward & Forward” method.

In pseudo-code (Algorithm 1), we identify the inputs of all of our products *P* by fixing a product $p_{i}$ ($p_{i}\in P$ where $1\le i\le No.\_of\_products$) and apply the “Backward” approach first (“$get\_n\_input\_candidates()$”). This gives us the top *n* input candidates *D* ($d_{j}\in D$ where $1\le j\le n$) for the input $p_{i}$ (we remove self-inputs (“$drop()$”)). Then, for each $d_{j}$, we apply the “Forward” approach (“$get\_n\_output\_candidates()$”) to identify the outputs of $d_{j}$, called *T*. We then look for product $p_{i}$ in the outputs (*T*) of *D*. If we find $p_{i}$ in *T*, we take the rank (“$getRank()$”) of $p_{i}$ in *T* and add it to the rank of $d_{j}$ in the inputs of $p_{i}$. And if we do not find $p_{i}$ in *T*, then we take the worst ranking which is the one of the last $p_{n}$ candidate product and add plus one, and add it to the rank of $d_{j}$ in the inputs of $p_{i}$. This technique updates (“$updateRank()$”) the initial ordering of $d_{j}$ as an input of $p_{i}$. We then order the products in ascending order (“$order\_by \_rank\_ascending()$”). This makes “2” the minimum and best possible rank meaning that $d_{j}$ was the first candidate (rank 1) as an input to $p_{i}$ and $p_{i}$ was the first candidate (rank 1) as an output to $d_{j}$. Further details on the fine-tuning of the parameters in the method can be found in the Additional file 1.

**Algorithm 1:**
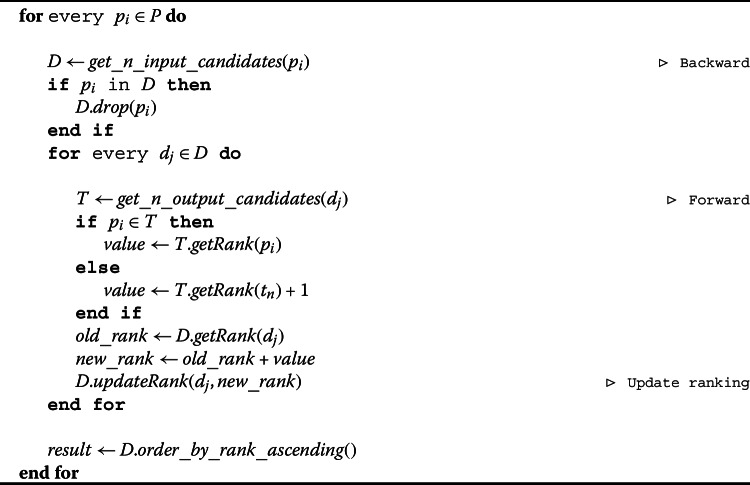
Backward & Forward Algorithm

By merging both the “Backward” and then the “Forward” approach, the combined method first identifies product candidates and then cross-verifies them through the Backward and Forward steps, thereby reducing noise and refining the input-output product results.

## Results

To produce our results, we apply the “Backward & Forward” method to HS4 and HS6 products, identifying the top three input candidates for each product. We limit the prediction to three inputs for two purposes: it ensures we go beyond a single input which captures cases where the correct input might rank second or third, and most importantly it keeps the manual validation process manageable by reducing the number of links requiring labeling.

**Figure 2:**
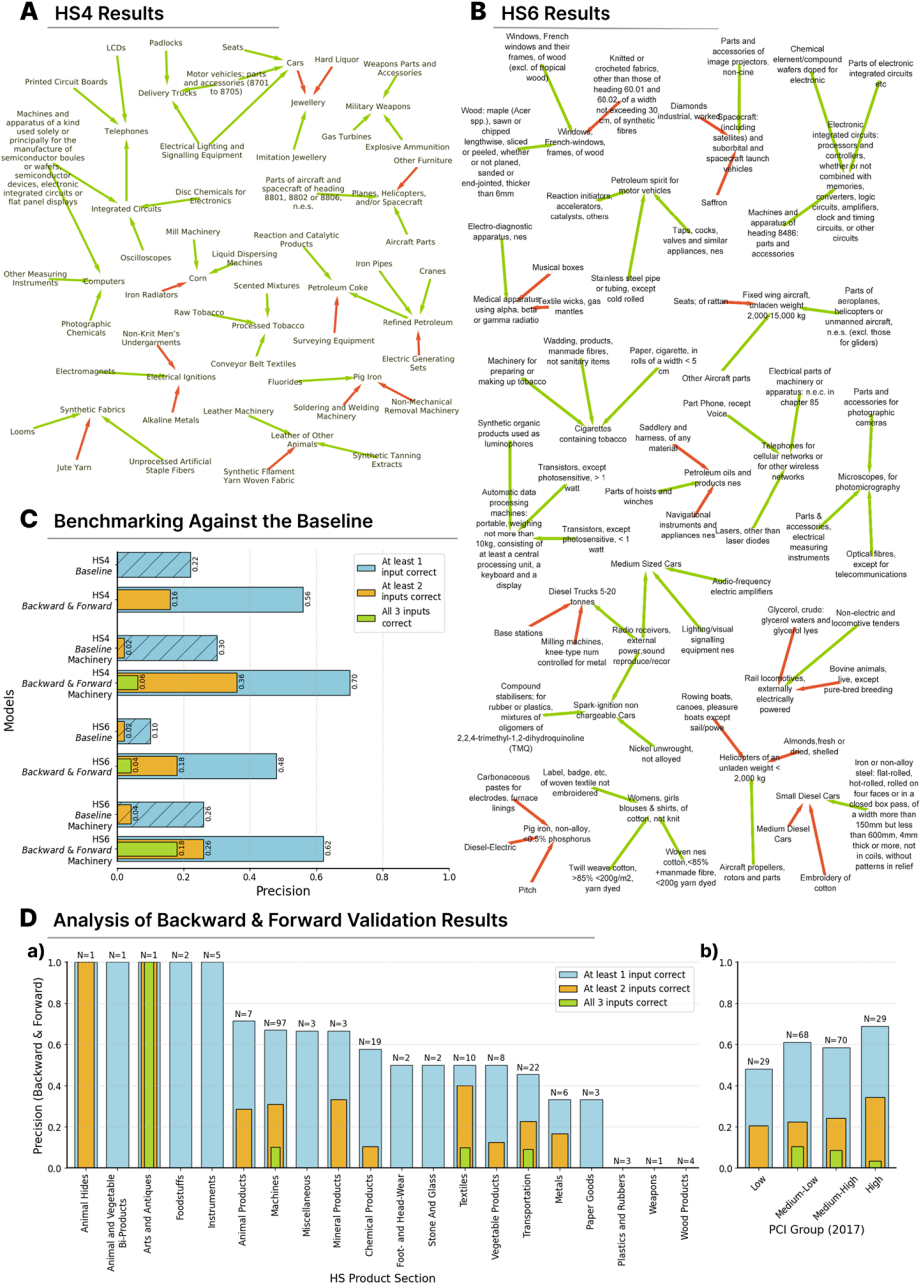
Subsets of our value chain network results, focusing on HS4 (A) and HS6 (B) products. Directed edges denote input-output connections, with red indicating misclassifications and green denoting correct identifications. (C) Bar charts comparing the performance of the Baseline with the Backward & Forward method, showing the percentage of one, two and three inputs correctly identified for 50 randomly sampled output products from each dataset (HS4, HS6, and Machinery products), with relations manually labeled to assess correctness. (D) Bar charts providing deeper insights into the Backward & Forward method’s performance, analyzing correct input identifications across HS Product Sections (a) and PCI groups (b) : Low ($PCI<-0.5$), Medium-Low($-0.5\leq PCI<0.5$), Medium-High ($0.5 \leq PCI<1.2$) and High ($PCI\geq 1.2$)

Our initial application of the “Backward & Forward” method focused on around 1200 HS4 products. In Fig. 2 (A) we see part of the value chain networks produced by the “Backward & Forward” method using HS4 trade data. These examples were validated manually for visualisation purposes. Red edges represent false positive, while the green edges are true positive value chain relationships.

Examples of accurately identified products are the inputs for “Cars”: “Motor vehicle parts and accessories (8701 to 8705)”, “Electrical Lighting and Signaling Equipment”, and “Seats”, while for “Delivery Trucks” the inputs are “Motor vehicle parts and accessories (8701 to 8705)”, “Electrical Lighting and Signaling Equipment”, and “Padlocks”. Other examples are “Telephones” and “Computers” where the inputs for the former are “LCDs”, “Printed Circuit Boards” and “Integrated Circuits”, and for the latter are “Photographic Chemicals”, “Machines and apparatus of a kind used solely or principally for the manufacture of semiconductor boules or wafers, semiconductor devices, electronic integrated circuits or flat panel displays” and “Other Measuring Instruments”. The other correct results we can see in the figure are for “Processed Tobacco”, “Integrated Circuits”, and “Military Weapons”.

However, we also see some false positive results in Table 1. In the example of “Electrical Ignitions” the model incorrectly predicts as inputs “Alkaline Metals” and “Non-Knit Men’s Undergarments” while it correctly predicts “Electromagnets”. Other examples where our model is able to identify only one correct input are “Jewellery” and “Pig Iron”.

**Table 1:**
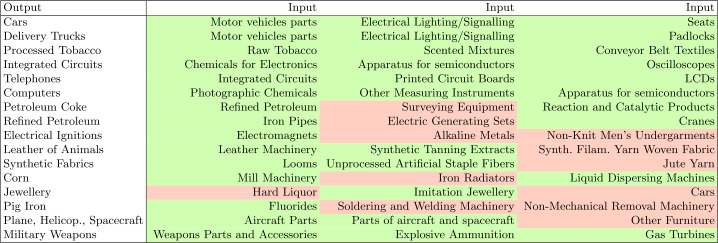
HS4 examples produced by the Backward & Forward method where the green cell represents a correctly predicted input candidate and red incorrectly. For readability, some of the product names have been shortened

Next, we applied our method to a more granular product classification, namely the HS6, which consists of over 5000+ unique products. In Fig. 2 (B) we see examples of the value chain networks produced by the “Backward & Forward” method using HS6 products. Notably, our method accurately identifies the inputs of “Medium Sized Cars”, “Cigarettes containing tobacco”, “Telephones for cellular networks or for other wireless networks” and “Electronic integrated circuits: processors and controllers, whether or not combined with memories, converters, logic circuits, amplifiers, clock and timing circuits, or other circuits” even at this more granular level of product classification.

The model predicted false positive results for “Helicopters of an unladen weight <2000 kg” with the inputs “Rowing boats, canoes, pleasure boats except sail/powe” and “Almonds, fresh or dried, shelled”. The product “Pig iron, non-alloy, <0.5% phosphorus” has no correct inputs.

### Validation

Due to the absence of a gold-standard true-positive input-output product dataset, we evaluate our “Backward & Forward” estimation of the $L_{p_{1}p_{2}} $ term by manually labeling four random samples, each consisting of 50 products. These are two random samples for HS4 products (one considering all products and another random sample considering only products in the machinery & transportation HS sections), as well as two random samples for HS6 products (also, one considering all products and another random sample considering only products in the machinery & transportation HS sections). For each of these random samples we choose three inputs, randomly to establish a null-model baseline, and then again with our method.

We label relationships as true only when they represent direct input-output relationships (e.g., vehicle parts as an input to car). Conversely, relationships are labeled as incorrect if the product is an indirect input (e.g., iron ore as an input to car) or not an input at all. We also acknowledge that as the classification level becomes more detailed (e.g., moving from HS4 to HS6), the process of manual labeling demands greater technical expertise. Despite our best efforts to ensure precision, this may introduce slight labeling inaccuracies.

Figure 2(C) shows the percentage of outputs having at least one, two and all three inputs correctly identified. On the y-axis we have the different methods: “Baseline Model” and “Backward & Forward Model” using the HS6 and HS4 product classifications. The “Backward & Forward” method successfully identifies at least one accurate input for over 40% of HS6 products (in total 5000+) and more than 56% of HS4 products (in total 1200+), marking a performance more than twice that of a random baseline model. Furthermore, “Backward & Forward” method can identify at least one of the inputs for 70% of the 50 HS4 products coming from the group of “Machinery” (machinery and transportation HS Section), whereas the baseline can identify only 30%. The random baseline also performs poorly when evaluated on the task of identifying more than one input correctly, when our model is able to identify two and sometimes three inputs correctly. While this validation shows that the accuracy of our model is far from perfect, it significantly–and substantially–beats the random benchmark, showing that the model is capturing information that is relevant to identify input-output relationships at the product level.

We further explore the accuracy of our method across different HS sections and levels of economic complexity groups. We find that the Backward & Forward method (Fig. 2 D (a)) successfully identifies all three inputs correctly in some products from the Machines, Textiles, and Transportation sectors, which also constitute the largest samples in our dataset. Additionally, the model performs well in the Arts and Antiques and Animal Hides sectors; however, due to the very small sample sizes in these cases, no definitive conclusions can be drawn. When considering sectors where the method most successfully identifies two inputs, the results, in descending order of performance, are: Textiles, Mineral Products, Machines, Animal Products, Transportation, Metals, Vegetable Products, and Chemical Products. In Fig. 2 D b) (based on 196 samples, as the 2017 PCI data for 4 products was unavailable), the Backward & Forward method demonstrated strong performance in identifying at least one or two inputs for products with high Product Complexity Index (PCI). However, the highest average precision for correctly predicting all three inputs was observed in products belonging to the Medium-Low and Medium-High PCI groups.

We believe we receive higher precision in the sample “Machinery” and on high-complexity products partly due to the data, which predominantly originates from countries with well-developed industrial sectors. By expanding the dataset with additional regional trade data from other countries, we expect this precision to increase further.

The performance of the “Backward & Forward” method is particularly significant, as no existing dataset offers input-output relationships at such a granular level of product classification (HS6 and HS4). We aspire for our model to serve as a foundational benchmark that future research can build upon. To support this, we are making our code and HS4 input-output results publicly available to ensure the reproducibility of our findings and to encourage the development of improved methodologies.

## Implications

Detailed product-level input-output data has several applications. Here, we focus on three. First, we use our proportional allocation model (Sect. 4, equation (2)) to estimate product-level trade flows between regions. Second, we explore the potential of our method going beyond the prediction of only three inputs. Third, we examine variations in the complexity of products along the value chain estimated according to the Product Complexity Index (PCI) (Hidalgo and Hausmann [18], Hausmann et al. [15], Hidalgo [16]).

For the first application, we must estimate $X_{r_{1} p_{1} r_{2} p_{2}}$ using equation (2). This represents the flow of product $p_{1}$ coming from region $r_{1}$ used by region $r_{2}$ to produce product $p_{2}$.

Consider the use of *engine parts* from *Jiangsu (China)* for the production of *cars* in *Barcelona (Spain)*. To estimate this flow we need to first estimate the share of *Jiangsu* in *Spain’s* import of Chinese *engine parts* ($X_{r_{1} p_{1} c_{2}}/\sum _{r_{1}} X_{r_{1} p_{1} c2}$), which is 16.7%. We also need to estimate the share of *cars* in *Barcelona’s* exports of products using engine parts as an input $X_{r_{2} p_{2}}/\sum _{p_{2}} X_{r_{2} p_{2}} L_{p_{1} p_{2}}$. Since our method to estimate $L_{p_{1} p_{2}}$ does not provide us with a full list of value chain relationships, we must bound this term. The bounds range from considering that all imports of engine parts are used for car production ($L_{p_{1} p_{2}}=1$ only if $p2=cars$) and considering that imports are allocated proportionally among all of Barcelona’s exports ($L_{p_{1} p_{2}}=1$ ∀ *p*2). In this example, the range of the share is 100% to 11.8%. Finally, we scale these fractions by Barcelona’s total imports of Chinese *engine parts*, which is 54.6*M* USD. Since in this case, $L_{p_{1} p_{2}} =1$, we can multiply these terms to estimate *Barcelona’s* imports of *Jiangsu’s* engine parts that are used to produce *car* exports. This results in an estimate in the range: 1.076*M* USD to 913*M* USD of *engine part* exports from *Jiangsu* for the production of *car* exports between 2017 and 2020 (Fig. 1).

**Figure 3:**
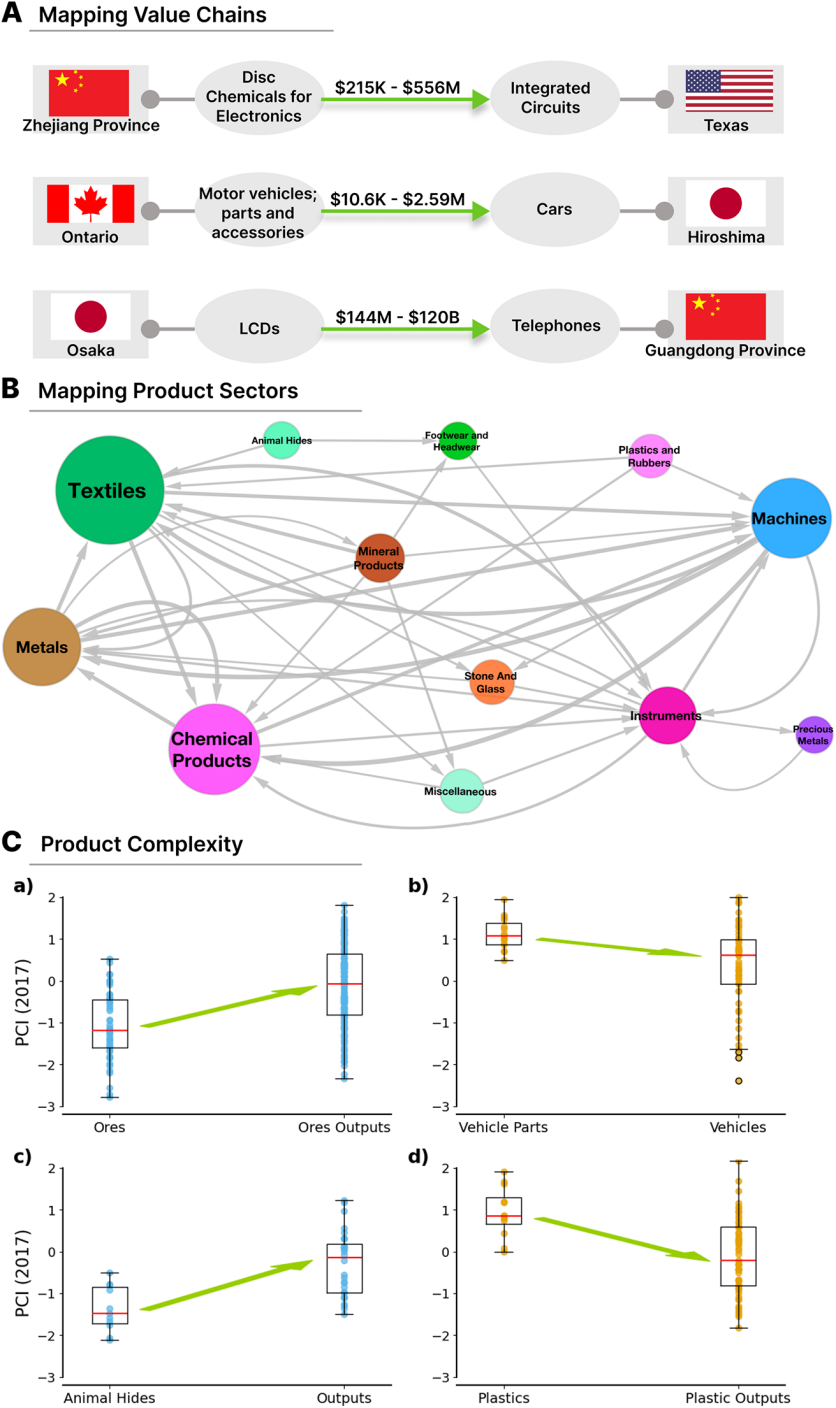
(A) Estimation of trade flow between two trading regions and two input-output products in the period between 2017 and 2020. (B) A subset of the input-output HS Sector product network, aggregated from HS4 input-output data. Node size represents the sample size of each sector, while edge weights are normalized to reflect the relative strength of connections. (C) Relationship between the Product Complexity Index and the Value Chain Position of products belonging to the categories of Ores (a), Vehicle Parts (b), Animal Hides (c), and Plastics (d) which are identified manually by their HS codes. The outputs for Vehicle Parts, Ores, Animal Hides, and Plastics are derived from the results obtained using the Backward & Forward method

Consider the trade flow of *LCDS* from *Osaka, Japan* used by *Guangdong, China* to export *Telephones*. *Osaka*’s share of Japanese *LCDS* imported by *Guangdong* is 33.8%, while *Guangdong*’s share of *Telephone* exports is 12.1%. With a 3.553*B* USD flow of *LCDS* from Japan to *Guangdong*, we estimate Guangdong imported between 145*M* and 120*B* USD of *LCDS* for the export of *telephones* between 2017 and 2020 (see Fig. 3(A)).

Using the same approach, we find that the trade flow of *disc chemicals for electronics* from *Zhejiang, China* to *Texas, United States* for the export of Integrated Circuits ranged from 215*K* USD to 556*M* USD, while *Hiroshima, Japan* imported between 10.6*K* USD and 2.59*M* USD worth of *motor vehicles; parts and accessories* from *Ontario, Canada* for the export of *cars* in the period between 2017 and 2020 (Fig. 3(A)).

In our second application, to broaden the scope of our analysis and achieve a more generalized understanding of input-output relationships, we expanded the prediction approach beyond the top-3 input candidates. Specifically, we constructed an HS4 input-output dataset in which the Backward & Forward method includes all input candidates with a rank lower or equal to 10. This extension allowed us to capture a more comprehensive view of the product network, resulting in a graph composed of 26 interconnected components. Collectively, these components include 55,932 input-output relationships between 1227 products.

Analyzing the structural properties of the network revealed that products such as Metalworking Transfer Machines, Other Knit Clothing Accessories, Tulles and Net Fabric, Other Plastic Products, and Copper Housewares had the highest betweenness centrality, highlighting their roles as key intermediaries. Metalworking Transfer Machines connect upstream metalworking operations to diverse downstream industries, underscoring their critical role in manufacturing. Other Knit Clothing Accessories and Tulles and Net Fabric link raw materials in the textile sector to downstream applications in fashion and apparel. Similarly, Copper Housewares and Other Plastic Products act as versatile connectors between raw material suppliers and a wide range of consumer and industrial markets.

Building on the analysis of the HS4 input-output network, Fig. 3B illustrates the input-output network, where the products are aggregated by their respective HS sectors (22 in total). The size of each node represents the sample size of HS4 products within the sector, while the edge weights are calculated as the number of input-output relationships between products from the two sectors divided by the sum of the total out-degree of the source node and the total in-degree of the target node. To enhance clarity, the visualization includes only the 41 highest-weight edges and their connected nodes, highlighting the most significant interdependencies.

The visualization highlights dominant flows such as the bidirectional interaction between Machines and Metals, emphasizing their central role in industrial production. Chemical Products demonstrate significant reliance on Textiles for essential inputs, while the dependency between Machines and Textiles reflects the pivotal role of advanced machinery in textile manufacturing. Other notable connections include Metals supplying Chemical Products, Chemicals playing a critical role in the production of Machines and Metals, and Metals contributing to Textiles through machinery and component materials. Secondary flows, such as Mineral Products feeding into Metals, and Textiles connecting with Instruments for precision manufacturing and quality control, further illustrate the intricate interdependencies that sustain sectoral interactions within the network.

This analysis highlights the potential of our method to go beyond the top three inputs, offering a deeper insight into the input-output relations between both HS4 and HS Sector product levels.

Our third application explores the complexity of products along the value chain. That is, whether the sophistication or knowledge intensity of a products grows, declines, or peak as we move along the value chain. For instance, are engine parts and LCDs more sophisticated products than finished cars or telephones?

Connecting value chains with product sophistication measures is important to those working in economic development since many classical and modern theories of economic development discuss diversification along value chains (e.g. from copper to electric wires) (Hirschman [19], Bontadini and Savona [6], Hidalgo [17], Rosenstein-Rodan [37]). Yet, since value chains can be explored in two directions, upstream and downstream, the question of which development path is more conducive to industrial upgrading requires understanding how value chain connections link products with different levels of sophistication. After all, development efforts attempt to move countries up the sophistication ladder.

During the last decade, this field was invigorated by the emergence of measures of product sophistication, extracted from international trade data, that can quantify the knowledge intensity of products (Hidalgo and Hausmann [18], Hausmann et al. [15], Hidalgo [16]). Yet, despite a few exceptions using aggregate data (Bahar et al. [3]), the connection between value chains and product sophistication has been rarely explored.

Using our data we can compare the average complexity of initial products (e.g. ores, animal hides, etc.), intermediate products (e.g. vehicle parts, plastics, etc.), and final products (e.g. vehicles, telephones), predicted using our “Backward & Forward” method.

Figure 3(C) shows the average complexity of products in a few selected categories (ores, animal hides, vehicle parts, and plastics) compared to their predicted outputs. Initial products that belong to the Harmonized System (HS) category of Mineral Products (HS2 code: 0526 and 0526), such as sand, clay, granite, cobalt ore, precious metal ore, have–on average–a lower product complexity index than their predicted outputs such as steel wire, nickel powder, and netting. Intermediate products in the category of vehicle parts (HS4 codes: 168412, 168413, 168414, 168482, 168483, 168484, 168487, 168501, 168501, 168502, 168503, 168504, 168505, 168506, 168507, 168512, 188706, 188708), such as electric motor, electric motor parts, transmissions, gaskets, have on average a higher product complexity index than their predicted outputs (e.g. motor vehicles, cars, delivery trucks, special purpose motor vehicles, etc.). We find a similar pattern for animal hides (primary products in HS category: 08) and intermediate plastic products (HS2 code: 0740). In the case of animal hides, the resulting outputs are of a greater complexity than the raw materials, while in the case of plastic products, the resulting outputs are of lower complexity.

These findings are consistent with the idea that complexity peaks in the middle of value chains, and that both primary products and finished goods are of lower complexity than intermediate inputs.

## Conclusion

Here we presented a first attempt to learn value chain relationships and estimate trade flows from trade data, by combining concepts from trade theory with machine learning techniques. While data on global value chains is notoriously aggregated, the “Backward & Forward” method offers a promising solution for mapping global value chains at the product level.

However, it is important to acknowledge that our method is not perfect. Although it operates at the product level, it may provide some false-positive value chain relationships, and it does not offer a complete input-output network. Additionally, optimizing the different parameters of our method can be a slow and complicated process.

Despite these limitations, the “Backward & Forward” method successfully identifies at least one accurate input for over 40% of HS6 products (in total 5000+) and more than 56% of HS4 products (in total 1200+), marking a performance more than twice that of a random baseline model. Moreover, our findings indicate that the method accurately identifies 70% of the first inputs for machinery products and correctly discerns three inputs for complex products like cars, integrated circuits, computers, and telephones. This validates the possibility of using international trade data at the regional level to identify value chain relationships. Furthermore, our model and results can serve as a foundational benchmark that subsequent research can refine and improve in the area of value chain mapping.

Increasing the accuracy of the “Backward & Forward” method represents an interesting avenue for future research. One approach is by fine-tuning the model with input-output tables that have a higher sectoral and geographical resolution than the present OECD ICIO data. Another approach is to expand the regional trade data by linking product codes with different classifications (e.g. Standard International Trade Classification (SITC), Central Product Classification (CPC), Standard Industrial Classification (SIC), Global Trade Analysis Project (GTAP)) to HS. Lastly, extending the validation of the method to more than three inputs per output would bring us closer to obtaining a complete value chain network.

## Supplementary Information

Below is the link to the electronic supplementary material.

(PDF 3.2 MB)

## Data Availability

The code, in the form of a Python notebook, is available in this folder. The notebook includes the implementation of the Proportional Allocation model using the Backward & Forward method, as well as the random baseline method. We also include in the folder the HS4 results in CSV form containing 3 inputs for every product. Please be aware that the trade data utilized for generating the results is not accessible to the general public, as it necessitates a Pro and Premium account on the Observatory of Economic Complexity (OEC) website.

## Abbreviations

U.S.: United States
U.K.: United Kingdom
USD: United States dollar
LCDs: Liquid Crystal Displays
OECD: Organisation for Economic Co-operation and Development
OEC: Observatory of Economic Complexity
ICIO: Inter-Country Input-Output
HS4: Harmonized System 4
HS6: Harmonized System 6
HS: Harmonized System
RCA: Revealed Comparative Advantage
PCI: Product Complexity Index
NIST: National Institute of Standards and Technology
SITC: Standard International Trade Classification
CPC: Central Product Classification
SIC: Standard Industrial Classification
GTAP: Global Trade Analysis Project
REA: EUROPEAN RESEARCH EXECUTIVE AGENCY
IAST: Institute For Advanced Study in Toulouse
ANR: French National Research Agency
