# Supplementary material for: Mapping global value chains at the product level
Source: EPJ Data Sci. 2025 Mar 12;14(1):21. doi: 10.1140/epjds/s13688-025-00521-5 (PMC11903633; doi:10.1140/epjds/s13688-025-00521-5)
Supplement: Supplementary file 1 — (PDF 3.2 MB) [file 13688_2025_521_MOESM1_ESM.pdf]

Mapping Global Value Chains at the Product Level

Appendix A OEC Subnational Data Manipulation

We use regional trade data from the Observatory of Economic Complexity (oec.world) for our results.
We combine the data for the 269 regions into a table containing as columns: "geography"− name of the region, "product"− HS4 product name, "value_imp"− the import trade value, "value_exp"− the export trade value, "geography_exp"− total exports of the region, "geography_imp"− total imports of the region, "product_exp"− total exports of the product, "product_imp"− total imports of the product, and two classical measures of specialization "rca_exp" and "rca_imp."

In the Table A1 we can see the final look of our trade data.

	geography	value_imp	product	geography_imp	product_imp	rca_imp	value_exp	geography_exp	product_exp	rca_exp
0	Ciudad de México	5882480.0	Other Vegetable Products	4.101108e+11	6.901484e+10	0.011240	1775336.0	3.633443e+11	1.509038e+09	1.057660
1	Guanajuato	2468431.0	Other Vegetable Products	3.754898e+10	6.901484e+10	0.051516	4320249.0	3.447602e+10	1.509038e+09	27.125367
2	Jalisco	9451035.0	Other Vegetable Products	9.338915e+10	6.901484e+10	0.079306	34593964.0	6.327343e+10	1.509038e+09	118.348546
3	Estado de México	508352.0	Other Vegetable Products	6.436369e+10	6.901484e+10	0.006189	2616971.0	3.181877e+10	1.509038e+09	17.803261
4	Nuevo León	3813095.0	Other Vegetable Products	1.256306e+11	6.901484e+10	0.023785	51550913.0	1.262054e+11	1.509038e+09	88.418374
...	...	...	...	...	...	...	...	...	...	...
196117	Metropolitana De Santiago	6178.30	Titanium Ore	2.233049e+11	1.166828e+11	0.000013	551658.00	2.108547e+11	2.649914e+08	3.225070
196118	Metropolitana De Santiago	172.95	Granulated Slag	2.233049e+11	3.079868e+08	0.000136	335812.32	2.108547e+11	4.000072e+10	0.013006
196119	Metropolitana De Santiago	848039.05	Prepared Explosives	2.233049e+11	1.898840e+09	0.108167	38389897.04	2.108547e+11	1.352253e+09	43.980460
196120	Metropolitana De Santiago	483473.53	Railroad Ties	2.233049e+11	4.331550e+09	0.027033	191154.59	2.108547e+11	7.041212e+08	0.420570
196121	Metropolitana De Santiago	274.74	Scrap Aluminium	2.233049e+11	5.365896e+10	0.000001	98316711.65	2.108547e+11	1.346654e+11	1.131025

Table A1 OEC Final Trade Data Table

Appendix B OECD International Data Manipulation

To fine-tune our "Backward and Forward" method we use the OECD Inter-Country Input-Output (ICIO) Tables. Here, we work with the intermediate use at basic prices data from 2016, 2017 and 2018, and we merge them by summing up the trade flows. To clean the data, we merge the data of China "CN1", "CN2" and "CHN" into "CHN". We do the same to the Mexico data, "MX1", "MX2" and "MEX" into "MEX". We also remove the country "ROW" which represents "Rest of the World" as it can introduce noise to the model.

Concerning the industries, we remove the industry 97T98 “Activities of households as employers; undifferentiated goods- and services-producing activities of households for own use” as it has not been traded.
We end up with a matrix of 2904x2904 industries-country pairs (44x66).
Moreover, we put the trade flow of reexports, export from and to the same country, to 0.

First, we start by creating the tunning table. For every country-industry pair, we put the country code in ”geography” column and the product code in ”product” column. Then in “value_imp” column we sum over the columns and for the “value_exp” we sum over the rows. Next, we calculate the “geography_exp” by grouping over “geography” and summing up the “value_exp”, and “product_exp” by grouping over ”product” and summing up the “value_exp”. We do the same to calculate the “geography_imp”, by grouping over “geography” and summing up the “value_imp”, and “product_imp” by grouping over “product” and summing up the “value_imp”. As we did for the Trade Data, using the formula $\frac{\frac{value\_exp}{geography\_exp}}{\frac{product\_exp}{sum(value_e xp)}}$ we calculate “rca_exp”. And we use the formula $\frac{\frac{value\_imp}{geography\_imp}}{\frac{product\_imp}{sum(value_i mp)}}$ to calculate “rca_imp”.
Table B2 shows the final look of the training data.

	value_imp	geography	product	geography_imp	product_imp	rca_imp	value_exp	geography_exp	product_exp	rca_exp
0	11619.407384	AUS	01T02	353818.854850	835032.99528	1.440590	22935.551244	595965.513523	768630.020805	1.834052
1	4840.318308	AUT	01T02	321604.777528	835032.99528	0.660220	2193.216844	349635.801449	768630.020805	0.298944
2	7049.627146	BEL	01T02	526307.471482	835032.99528	0.587576	4927.290870	532869.434581	768630.020805	0.440667
3	27917.032198	CAN	01T02	876406.697376	835032.99528	1.397336	37023.829481	909091.443344	768630.020805	1.940874
4	3671.147871	CHL	01T02	93649.791607	835032.99528	1.719620	7221.641047	174677.439401	768630.020805	1.970256
...	...	...	...	...	...	...	...	...	...	...
2899	521.915500	ZAF	94T96	147558.428838	253890.016018	0.510307	1.420904	158225.011733	15681.17867	0.020977
2900	2375.654280	TWN	94T96	576254.654859	253890.016018	0.594791	93.548425	693550.512370	15681.17867	0.315080
2901	4563.273980	THA	94T96	473645.715393	253890.016018	1.390011	0.000000	396945.386345	15681.17867	0.000000
2902	41.091241	TUN	94T96	29409.824719	253890.016018	0.201582	6.295216	21764.013076	15681.17867	0.675669
2903	1962.493248	VNM	94T96	502262.634668	253890.016018	0.563732	1.248322	345511.049333	15681.17867	0.008440

Table B2 Final OECD Table

Then, we move on to creating the labeled data. For this, we take the initial table (country-industry x country-industry pairs and we sum over the industries so that we get a matrix of 44x44 industries with their corresponding trade flows.
Afterwards, we apply the trade intensity formula for every input-output industry and get the Table B3. Trade Intensity (TI). TI index, according to Yamazawa[**?**], is used to determine whether the value of trade between two countries is greater or smaller than would be expected on the basis of their importance in world trade. It is defined as the share of one country’s exports going to a partner divided by the share of world exports going to the partner.
In our situation we will be using the TI between products (industries).

We calculate it as:

$$TI_{pp'} = \frac{X_{pp'} / \sum_{p'} X_{pp'}}{\sum_{p} X_{pp'} / \sum_{pp'} X_{pp'}} \tag{B1}$$

Where $X_{pp'}$ and $\sum_p X_{pp'}$ are the trade flows from industry p's and from all the industries to industry p', and where $\sum_{p'} X_{pp'}$ and $\sum_{pp'} X_{pp'}$ are industry p's total exports and total export of all industries respectively. An index of more (less) than one indicates a trade flow that is larger (smaller) than expected between the two products (industries).

Then, we binarize the matrix by applying

$$\begin{cases} 1, & \text{if } TI \geq 1 \\ 0, & \text{otherwise} \end{cases} \tag{B2}$$

The labeled data that we get is depicted in Table B3.

When we map the entire input-output network between the industries we get the value chain shown in the Figure B1.

	01T02	03	05T06	07T08	09	...	84	85	86T88	90T93	94T96
01T02	1	1	0	0	0	...	1	1	1	1	1
03	0	1	0	0	0	...	1	1	1	1	1
05T06	0	1	1	1	1	...	1	1	1	1	1
...	...	...	...	...	...	...	...	...	...	...	...
86T88	0	0	0	0	0	...	0	1	1	0	1
90T93	0	0	0	0	0	...	1	1	1	1	1
94T96	0	0	0	0	0	...	0	1	0	0	1

Table B3 Trade Intensity Binary OECD Table

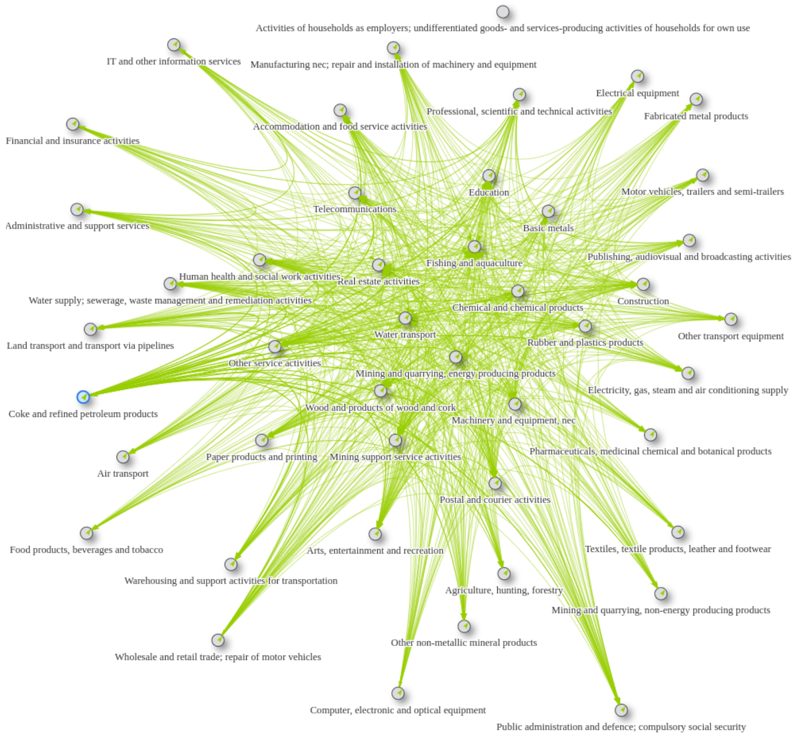

Fig. B1 Depicts the full OECD binarized/labeled network that consists of nodes representing sectors and directed edges representing the input-output connections between the sectors.

B.1 Fine-tuning

In the "Backward & Forward" method we come across a few parameters that require optimizing: two parameters in the "Backward" and two in the "Forward" approach.

In the "Backward" approach we have the specialization of exporters threshold ($rca\_locations\_1$). This is the minimum RCA^{export} a location needs to have to be considered specialized in the export of a certain product. Then, we have the import specialization of industries/products threshold ($rca\_industries\_1$). This helps us rank the input candidates by counting how many of the specialized export locations, that we identified before, are also specialized in the import of the input candidate.

Similarly, in the "Forward" approach, we have the specialization of importers threshold ($rca\_locations\_2$). This is the minimum RCA^{import} a location needs to have to be considered specialized in the import of a certain product. Then, we have the export specialization of industries/products threshold ($rca\_industries\_2$). This helps us rank the output candidates by counting how many of the specialized import locations, that we identified before, are also specialized in the export of the output

candidate.

To optimize the parameters $rca\_locations\_1$, $rca\_industries\_1$, $rca\_locations\_2$ and $rca\_industries\_2$ we feed the OECD ICIO specialization data to the "Backward & Forward" Model. Then we use the OECD labeled data to evaluate the model.

Considering the nature of our value chain problem, the full result would be a sparse matrix with many 0s and very few 1s. Meaning that a big part of all the possible product pairs' input-output relationships (1488400 HS4 and 34692100 HS6 products) will be false and very few will be true. The model should prioritize identifying the true relationships. For this reason, we use precision as an evaluation metric which tells us how many of our predicted input-output relationships are correctly classified [**?**].

To optimize the parameters we perform a grid search. Looking at the distributions of RCAs in Figure B2, we test all the combinations of $rca\_locations\_1$, $rca\_industries\_1$, $rca\_locations\_2$ and $rca\_industries\_2$ assigned with a value in the interval of $[1, 6[$ with step of 0.5. For every combination, we calculate the corresponding precision.

The Figure B2 shows the 500 combinations that achieved the highest precision. The best precision is 0.67 with the $rca\_locations\_1 = 2$, $rca\_industries\_1 = 1.5$, $rca\_locations\_2 = 3.5$ and $rca\_industries\_2 = 2$.

In Figure B2 we can see the value chain network produced by the "Backward & Forward" model using the best parameters. With the OECD labeled data, we are able to colour the correct links with green and the incorrect ones with red. There are 84 true-positive and 48 false-positive input-output links.

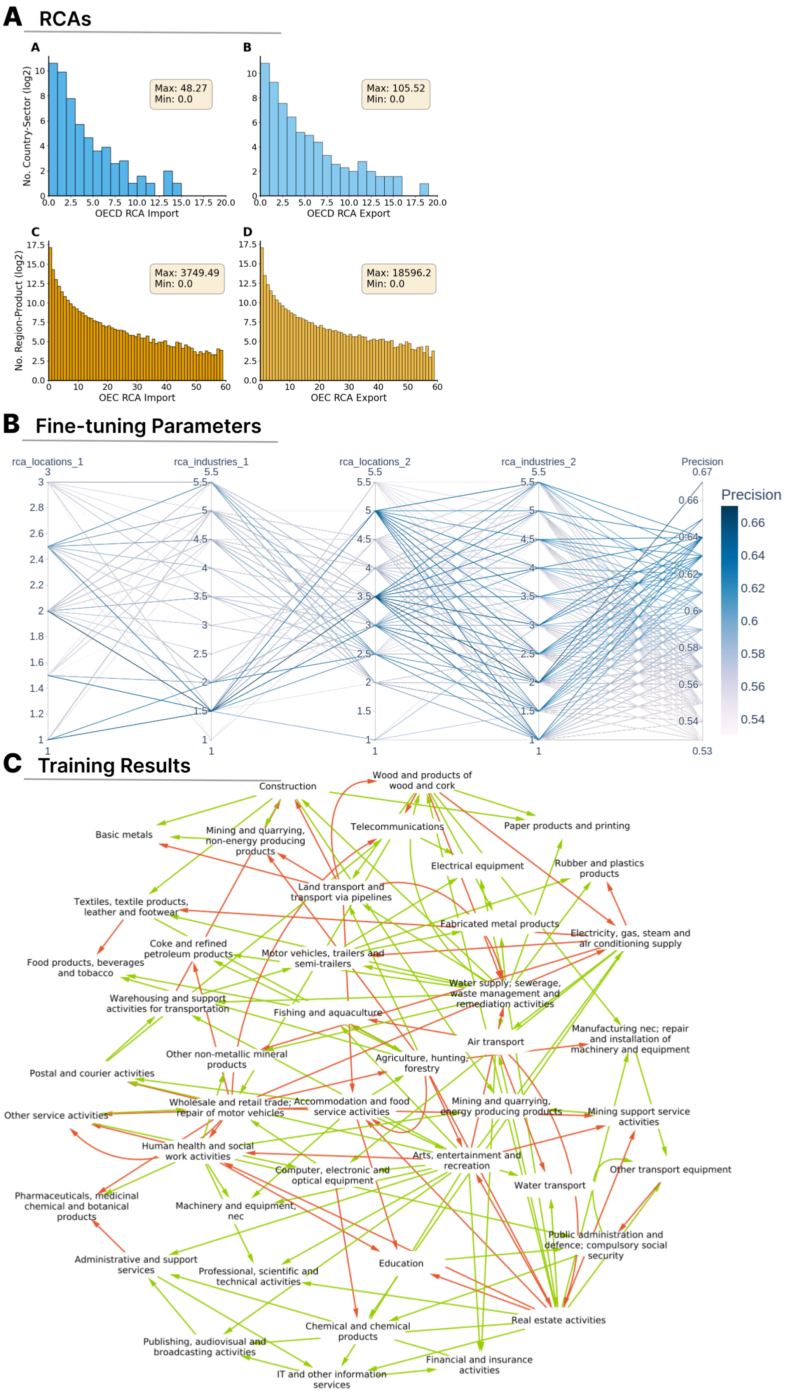

Fig. B2 (A) Total OECD RCA import (a), OECD RCA export (b), OEC RCA import (c) and OEC RCA export (d) distribution across all the product-region in the case of the OEC and the sector-country pairs in the case of OECD data between 2017 and 2020. (B) Parallel coordinate plot showing the 500 highest in precision combinations of the parameters: rca_location_1, rca_industries_1, rca_location_2 and rca_industries_2. The precision values are rounded to two decimals. (C) Resultant value chain network that consists of nodes representing industries and directed edges representing the input-output connections between the industries. The edges are labelled using the OECD labelled data, with red edges indicating misclassified links and green edges indicating correctly identified inputs by the Backward & Forward method.
